# Metabolic insights into phosphofructokinase inhibition in bloodstream-form trypanosomes

**DOI:** 10.3389/fcimb.2023.1129791

**Published:** 2023-02-14

**Authors:** Zandile Nare, Tessa Moses, Karl Burgess, Achim Schnaufer, Malcolm D. Walkinshaw, Paul A. M. Michels

**Affiliations:** ^1^Institute of Immunology and Infection Research, School of Biological Sciences, Ashworth Building, The University of Edinburgh, Edinburgh, United Kingdom; ^2^EdinOmics, RRID:SCR_021838, Centre for Engineering Biology, School of Biological Sciences, CH Waddington Building, The University of Edinburgh, Edinburgh, United Kingdom; ^3^Institute of Quantitative Biology, Biochemistry and Biotechnology, School of Biological Sciences, CH Waddington Building, The University of Edinburgh, Edinburgh, United Kingdom; ^4^Wellcome Centre for Cell Biology, School of Biological Sciences, Michael Swann Building, The University of Edinburgh, Edinburgh, United Kingdom

**Keywords:** Trypanosoma, phosphofructokinase, mode of inhibition, metabolomics, glycolytic metabolites, ATP production, carnitine/acylcarnitine translocation

## Abstract

Previously, we reported the development of novel small molecules that are potent inhibitors of the glycolytic enzyme phosphofructokinase (PFK) of *Trypanosoma brucei* and related protists responsible for serious diseases in humans and domestic animals. Cultured bloodstream-form trypanosomes, which are fully reliant on glycolysis for their ATP production, are rapidly killed at submicromolar concentrations of these compounds, which have no effect on the activity of human PFKs and human cells. Single-day oral dosing cures stage 1 human trypanosomiasis in an animal model. Here we analyze changes in the metabolome of cultured trypanosomes during the first hour after addition of a selected PFK inhibitor, CTCB405. The ATP level of *T. brucei* drops quickly followed by a partial increase. Already within the first five minutes after dosing, an increase is observed in the amount of fructose 6-phosphate, the metabolite just upstream of the PFK reaction, while intracellular levels of the downstream glycolytic metabolites phosphoenolpyruvate and pyruvate show an increase and decrease, respectively. Intriguingly, a decrease in the level of O-acetylcarnitine and an increase in the amount of L-carnitine were observed. Likely explanations for these metabolomic changes are provided based on existing knowledge of the trypanosome’s compartmentalized metabolic network and kinetic properties of its enzymes. Other major changes in the metabolome concerned glycerophospholipids, however, there was no consistent pattern of increase or decrease upon treatment. CTCB405 treatment caused less prominent changes in the metabolome of bloodstream-form *Trypanosoma congolense*, a ruminant parasite. This agrees with the fact that it has a more elaborate glucose catabolic network with a considerably lower glucose consumption rate than bloodstream-form *T. brucei*.

## Introduction

Different subspecies of the protist *Trypanosoma brucei* cause Human African Trypanosomiasis (HAT), also known as ‘sleeping sickness’ and, together with other *Trypanosoma* species such as *T. congolense*, can cause similar diseases in domestic animals in sub-Saharan Africa (Giordani et al., 2016; Büscher et al., 2017). The parasites, after having been transmitted between mammalian hosts by the bite of an infected tsetse fly, enter the bloodstream and can subsequently invade extravascular niches of different tissues, including the brain (Morrison et al., 2023). When proliferating in the blood as long-slender forms, *T. brucei* cells are entirely dependent on the abundantly available glucose for their ATP production. The tricarboxylic acid (TCA) cycle enzymes and oxidative-phosphorylation system of the trypanosome’s single large mitochondrion are severely repressed in its bloodstream-form (BSF), and all ATP production occurs essentially by substrate-level phosphorylation with most of the end-product of glycolysis, pyruvate, excreted from the cells (reviewed in Michels et al., 2021).

A unique property of the group of protists to which trypanosomatids belong is the compartmentalization of most glycolytic enzymes, from hexokinase to phosphoglycerate kinase, within multiple small peroxisome-related organelles called glycosomes (Opperdoes and Borst, 1977). Only the last three enzymes of the pathway, including pyruvate kinase responsible for the net production of ATP, are present in the cytosol (**Supplementary Figure 1**).

In addition to pyruvate, small amounts of other products may be formed from glucose, including succinate, alanine, and acetate (Mazet et al., 2013). Some pyruvate may enter the mitochondrion where it is oxidized and decarboxylated by the pyruvate dehydrogenase complex resulting in the formation of acetyl-CoA. This glucose-derived acetyl-CoA, as well as acetyl-CoA that may be formed within the mitochondrion from threonine, can then be converted *via* acetyl-phosphate into acetate with concomitant production of ATP by the cyclic activity of acetate:succinate CoA-transferase (ASCT) and succinyl-CoA synthetase (SCS) (Van Hellemond et al., 1998). Intramitochondrial ATP is required to maintain the essential electrochemical proton gradient across the inner mitochondrial membrane by the F_o_F_1_-ATP synthase acting in its reverse mode (*i.e*., hydrolyzing ATP to create the proton gradient) (Nolan and Voorheis, 1992; Vercesi et al., 1992; Schnaufer et al., 2005; Mochizucki et al., 2020). Alternatively, the proton gradient may be generated from glycolytically produced ATP that has been imported by the ATP/ADP carrier (**Supplementary Figure 1**). To what extent each source contributes to intramitochondrial ATP under physiological conditions in proliferating long slender forms is presently unclear and may depend on the host tissue where the parasite resides and on the carbon source(s) available (discussed in Michels et al., 2021). The mitochondrially produced acetate has been shown to be essential for fatty-acid synthesis. Acetate is exported from the mitochondrion through a hypothetical acetate shuttle and converted again to acetyl-CoA in the cytosol by a cytosolic AMP-forming acetyl-CoA synthetase, an enzyme that was shown to be essential for the trypanosomes (Mazet et al., 2013).

Although glycolysis is also essential for BSF *T. congolense*, its rate of glucose consumption is considerably lower than that in BSF *T. brucei*. This may be related to the relatively low glucose concentration in blood of their ruminant hosts (2 – 4 mM compared to approximately 5.5 mM in human blood) (Cozzi et al., 2011; Mair et al., 2016). Moreover, it has a more elaborate glucose catabolic network, with an upregulated glycosomal succinate-producing pathway and an increased rate of ATP production in the mitochondrion by the conversion of pyruvate-derived acetyl-CoA to acetate through the ASCT/SCS cycle (**Supplementary Figure 1**) (Steketee et al., 2021).

Previously, we reported the development of novel small molecule inhibitors (‘CTCB compounds’) of *T. brucei* phosphofructokinase (PFK), the glycolytic enzyme that catalyzes the ATP-dependent phosphorylation of fructose 6-phosphate (F6P) to fructose 1,6-bisphosphate (F1,6BP). Addition of the inhibitor causes a block of the BSF trypanosomes’ glycolytic flux, leading to very fast parasite killing (Brimacombe et al., 2013; McNae et al., 2021). The compounds are highly selective for the PFK of trypanosomes and related protists; they bind in an allosteric pocket near the active site that regulates the switch between the T- and R-states (McNae et al., 2021). The pocket is not present in mammalian PFKs and, therefore, the compounds have no effect on the activity of human PFKs. Single-day oral dosing cures stage 1 disease in an animal model of HAT when the parasites proliferate in the blood. Furthermore, the compounds can cross the blood-brain barrier and cause important reduction of parasitemia in the brain (stage 2 HAT). Here we describe the results of our targeted analysis of metabolic changes caused by PFK inhibition by one of the most potent compounds, CTCB405, in cultured BSF *T. brucei*. Synthesis, chemical and pharmacokinetic properties of CTCB405, or 1-[(3,4-dichlorophenyl)methyl]-5-[2-(dimethylamino)ethyl] pyrrolo[3,2-c]pyridin-4-one, have been described by McNae et al. (2021). It inhibits purified recombinant PFK of the subspecies *Trypanosoma brucei brucei* Lister 427 with an IC_50_ = 0.18 ± 0.03 μM and kills *in vitro* cultured BSF trypanosomes of this subspecies with an EC_50_ = 0.37 ± 0.03 μM. In addition to analyzing the effect of CTCB405 on the metabolome of *T. brucei* Lister 427, we also studied a different, pleiomorphic *T. brucei* strain, EATRO 1125, and a *T. congolense* strain.

## Materials and methods

### Trypanosoma strains

Bloodstream-form (BSF) cells of the following two *Trypanosoma brucei brucei* strains were used: monomorphic strain Lister 427, single marker cell line (Wirtz et al., 1999), and pleiomorphic EATRO 1125 AnTat 1.1 cell line 90:13 (Engstler and Boshart, 2004). In addition, BSF cells of strain IL3000 of the species *Trypanosoma congolense* (Bienen et al., 1991), an important causative agent of the livestock disease nagana, were also used. The culturing of the different *Trypanosoma* strains is described in Supplementary Material section 1.1.

### Trypanosome motility measurements

To assess the effect of phosphofructokinase (PFK) inhibition on their energy metabolism and viability, aliquots of 990 μl of exponentially growing trypanosomes were taken from the culture flask at a density of ~9 x 10^5^ cells/ml and transferred to wells of a 48-well culture plate (Corning). The number of motile cells was determined using a hemocytometer. Subsequently, 10 μl of PFK inhibitor CTCB405, appropriately diluted in 100% DMSO (McNae et al., 2021), was added to each suspension to give final concentrations of 0, 0.25, 0.5 and 0.75 μM. The number of motile cells was determined at 0, 5, 10, 15, 25, 45 and 60 min after addition of CTCB405. Two technical replicates were performed for each trypanosome cell line, and the data plotted as number versus time in GraphPad Prism (version 9.0).

### Determination of ATP content of trypanosomes

The time-dependent effect of CTCB405 on the ATP content of trypanosomes was assessed using the CellTiter® Glo 3D kit (Promega). This kit has been developed for viability assessment of cells and was previously used to estimate the time by which trypanosomes are killed by PFK inhibitors and known trypanocidal drugs (McNae et al., 2021). In the work described in this paper, trypanosomes were grown to a density of 9.5 x 10^5^ cells/ml in 50 ml in 150 cm^2^ surface vented tissue culture flasks (Corning) prior to inhibitor treatment. Incubation of the cultures was done at 37°C under water-saturated air with 5% CO_2_ for different periods of time after addition of CTCB405 (0, 5, 10, 15, 25 45 and 60 min), with four biological replicates, before taking samples of 100 μl from each flask. CTCB concentrations used were 0.25 μM for *T. brucei* Lister 427, and 0.75 μM for both *T. brucei* EATRO 1125 and *T. congolense* IL3000. Cultures were treated in reverse order (*i.e*., from 60 min down to 0 min) to ensure that all samples reached the endpoint at the same time and were processed in unison. Next, 100 μl samples were transferred to an opaque white 96-well plate (Greiner Inc) and an equivalent volume of CellTiter® Glo 3D reagent added to lyse trypanosomes. The plate was covered in foil and incubated on a rotary shaker for 5 min to aid cell lysis. Subsequently, the plate was incubated at room temperature for a further 20 min and the luminescence signal measured at a wavelength of 580 nm using a FLUOstar Omega microplate fluorescence scanner (BMG Labtech). The percentage change in ATP content in each sample was plotted and normalized to the untreated (t=0 min) cells. The ATP was estimated by normalizing to the total number of cells and using a standard curve prepared with serial dilutions of ATP (0 – 10 μM; Sigma-Aldrich) prepared in relevant sterile culture medium for each trypanosome strain. The remaining cultures, after samples had been taken, were quickly cooled on ice for metabolome extraction.

### Metabolomic profiling of trypanosomes

Liquid chromatography ion mobility mass spectrometry (LC-IM-MS) was used to follow the time-dependent metabolic changes caused in the trypanosomes upon administration of compound CTCB405. A detailed description of the methodologies used for metabolite extraction, data acquisition, processing and statistical analysis is provided in Supplementary Material section 1.2.

## Results

We first determined appropriate sublethal PFK inhibitor concentrations to be used for our metabolomic analysis. To that end, the effect of different CTCB405 concentrations on trypanosome mobility was assayed, based on prior knowledge that arrest of glycolysis in BSF trypanosomes by glucose depletion or inhibition of glucose uptake very rapidly affects flagellar beating resulting in arrest of mobility, preceding cell death (Ter Kuile and Opperdoes, 1991). **Supplementary Figure 2** shows the time-dependent reduction of motility, determined as described in the Materials and methods section, at four different CTCB405 concentrations for each of the three *Trypanosoma* strains. *T. b. brucei* Lister 427 cells showed the highest susceptibility to the inhibitor; *T. b. brucei* EATRO 1125 and *T. congolense* IL3000 required about three times more compound to reach a similar effect. Incubation with the higher concentration of inhibitor led to morphological changes such as shriveling up of some cells during the 1-h period, but no evidence for cell lysis was observed. The different susceptibility of the two *T. brucei* strains, which have 100% identical PFK sequences, is likely due to differences in the extent of inhibitor accumulation, as different doses are required to achieve similar intracellular levels (see below). However, possible variations in PFK expression and/or posttranslational modifications may also contribute to the different susceptibility observed. The PFK sequences of *T. brucei* and *T. congolense* share 87.7% overall identity, and a *T. congolense* PFK model structure generated by AlphaFold (Jumper et al., 2021) fits the *T. brucei* PFK crystal structure complexed with CTCB405, as determined by McNae et al. (2021), very well with an RMS value of 0.56 Å. All residues in the inhibitor-binding pocket are conserved between the enzymes of the two species (**Supplementary Figure 3**). Comparable effects on motility were achieved with 0.25 μM CTCB405 for *T. b. brucei* Lister 427 cells and 0.75 μM for *T. b. brucei* EATRO 1125 and *T. congolense* IL3000 cells, respectively, with the number of motile cells gradually decreasing by a factor of ~3-5 within one hour. These concentrations were chosen for use in subsequent metabolomic experiments.

**Figure 1:**
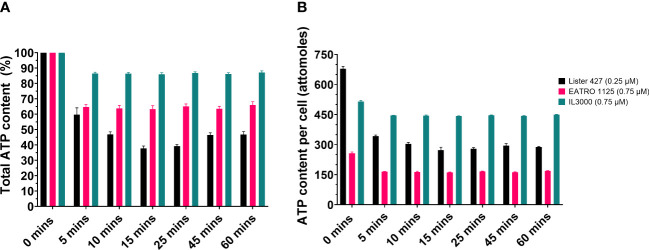
CTCB405 reduces the total ATP content in BSF *T. b. brucei* Lister 427, *T. b. brucei* EATRO 1125 and *T. congolense* IL3000 cells. Cell suspensions (9 x 10^5^ cells/ml) were treated with CTCB405 (0.25 μM for Lister 427, 0.75 μM for EATRO 1125 and IL3000) for different periods of time. **(A)** The relative ATP content of cells treated for various periods of time with PFK inhibitor, measured with the CellTiter-Glo® 3D Assay (Promega). ATP level of untreated cells (0 min) = 100%. The figure presents means ± standard deviations of four biological replicates of each *Trypanosoma* strain. **(B)** Estimation of the ATP content per cell by normalizing the data from panel A to the total number of cells per sample. An ATP standard curve was created to confirm that ATP content detection in the CellTiter-Glo® 3D Assay is linear (**Supplementary Figure 4**).

First, the effect of CTCB treatment on the total cellular ATP content was determined. Trypanosomes were treated with the selected compound concentration and the ATP content determined at seven time points during a 1-h incubation using the CellTiter® Glo 3D kit (Promega) as described in the Materials and methods section. This method measures the total amount of ATP liberated from cells after lysing them with reagent, thus not distinguishing between cytosolic, mitochondrial and glycosomal pools. An ATP standard curve was prepared to ascertain a linear relationship between the ATP concentration and the luminescence signal it generates by reacting with the assay reagent (**Supplementary Figure 4**). **Figure 1A** shows that CTCB405 reduced the ATP content in the three cell lines, albeit to varying degrees. In *T. b. brucei* Lister 427 the ATP level dropped in 5 min to approximately 60%, followed by a further decrease to approximately 38% after 15 min, before a slight increase during the remaining period. In comparison, the ATP content of *T. b. brucei* EATRO 1125 was reduced to about 65% within 5 min but remained relatively stable at the remaining time points. The ATP content of *T. congolense* IL3000 cells fell only to about 86% after 5 min and remained at that level for the remaining time course. **Figure 1B** presents an estimation of the ATP content per cell after normalizing to the total number of cells per sample.

**Figure 2:**
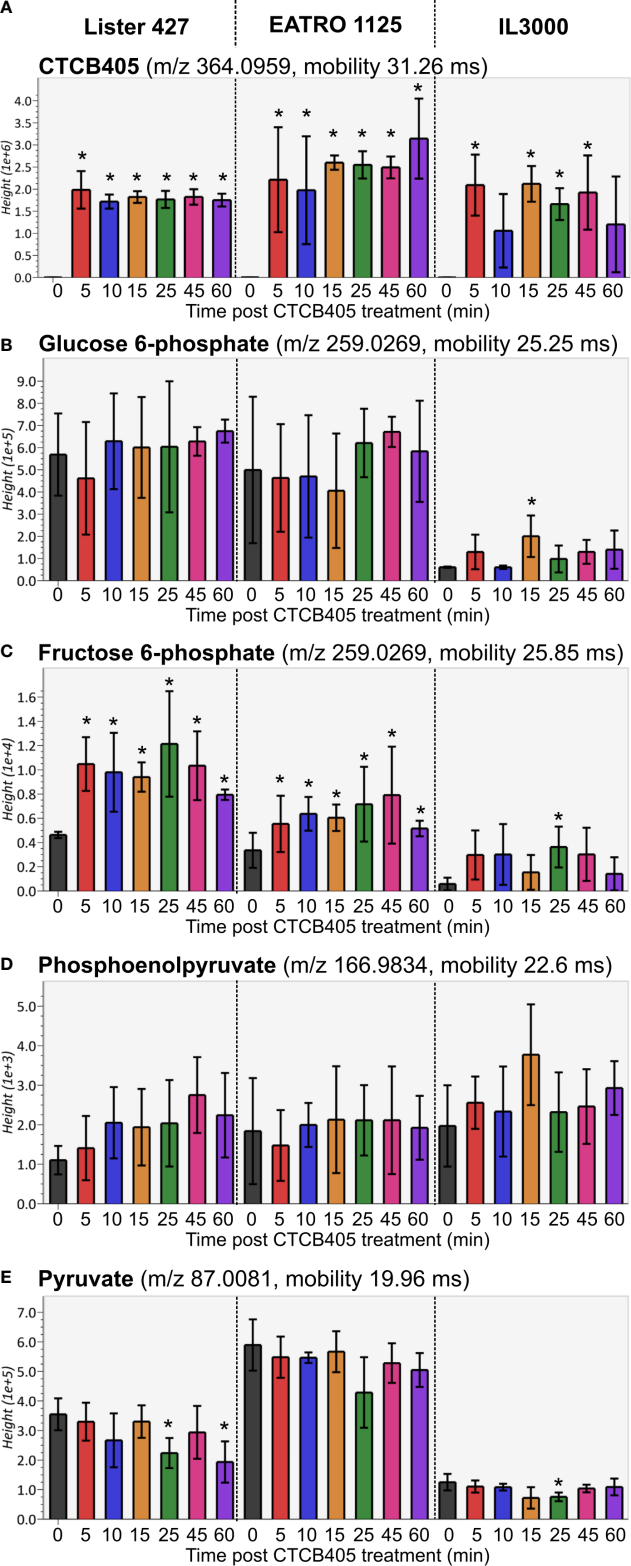
Relative amounts of selected glycolytic intermediates after CTCB405 treatment. Average peak height (Y-axis shows the absolute ion intensity in the drift tube of the instrument, accounting for accurate mass as m/z and drift time mobility in ms of each metabolite) and standard deviation (n = 4) for 0.25 μM CTCB405 treated BSF *T. b. brucei* Lister 427, and 0.75 μM CTCB405 treated *T. b. brucei* EATRO 1125 and *T. congolense* IL3000 cells are plotted over time for **(A)** CTCB405, **(B)** glucose 6-phosphate, **(C)** fructose 6-phosphate, **(D)** phosphoenolpyruvate, and **(E)** pyruvate. Cells were treated with CTCB405 for 1 h and samples collected at 0, 5, 10, 15, 25, 45 and 60 min post treatment. Glucose catabolites not found in the dataset (**Supplementary Table S1**): fructose 1,6-bisphosphate, glyceraldehyde 3-phosphate, 1,3-bisphosphoglycerate, 3-phosphoglycerate, 2-phosphoglycerate, and acetyl-CoA. Statistically significant (p-value ≤ 0.05) values compared for each time point to 0 min are indicated with an asterisk (*).

Subsequently, metabolomic analyses of all three cell lines were performed. The full metabolomics dataset is provided as **Supplementary Table 1**. Rapid accumulation of CTCB405 was observed in the cells (**Figure 2A**). CTCB405 was not detected in untreated (0 min) cells, but readily taken up within 5 min by cells of all three strains. The intracellular levels of CTCB405 remained relatively stable up to 60 min in *T. b. brucei* Lister 427 and EATRO 1125 cells, in contrast to *T. congolense* IL3000 cells, where levels slightly decreased over time, perhaps suggesting a less efficient internalization or more efficient turnover or excretion by these cells. In line with the proposed mechanism, inhibition of PFK by CTCB405 led to changes in the levels of various glycolytic intermediates (**Supplementary Table 2** and **Figure 2**). A rapid, significant increase (from 5 min) in intracellular levels of F6P, the substrate of PFK, occurred in all cell lines (**Figure 2C**). Furthermore, low amounts of intracellular phosphoenolpyruvate (PEP) with an increasing trend (**Figure 2D**), and pyruvate with a decreasing trend over time (**Figure 2E**) were observed, most notable in *T. b. brucei* Lister 427 and to a lesser extent in the other cell lines. It is worth noting that the amounts of F6P, its isomer G6P, and pyruvate were lower in *T. congolense* compared to the *T. b. brucei* strains (**Figures 2B, C, E**). The slightly increased levels of PEP and decreased levels of pyruvate may be due to a decreased level of F1,6BP, an allosteric activator of many pyruvate kinases, including those of trypanosomatids (Callens et al., 1991; Callens and Opperdoes, 1992; Morgan et al., 2014). Although this metabolite was not detected in the dataset, as the direct product of the PFK reaction its level was likely decreased by the PFK inhibition.

**Figure 3:**
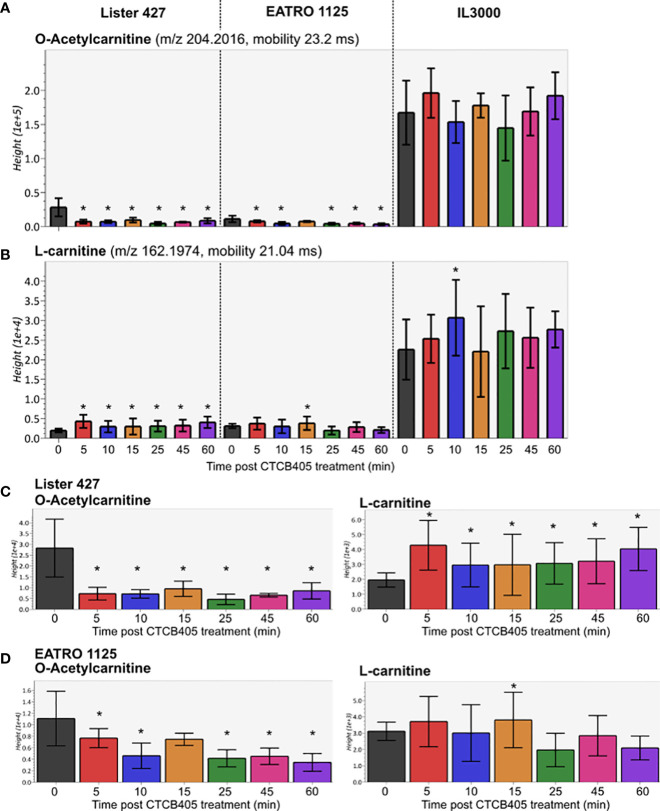
Relative amounts of O-acetylcarnitine and L-carnitine after CTCB405 treatment. Average peak height (Y-axis shows the absolute ion intensity in the drift tube of the instrument, accounting for accurate mass as m/z and drift time mobility in ms of each metabolite) and standard deviation (n = 4) for 0.25 μM CTCB405 treated BSF *T. b. brucei* Lister 427, and 0.75 μM CTCB405 treated *T. b. brucei* EATRO 1125 and *T. congolense* IL3000 cells are plotted over time for O-acetylcarnitine **(A)** and L-carnitine **(B)**. *T. b. brucei* Lister 427 **(C)** and EATRO 1125 **(D)** cells with adjusted Y-axis show changing levels of O-acetylcarnitine and L-carnitine over time. Cells were treated with CTCB405 for 1 h and samples collected at 0, 5, 10, 15, 25, 45 and 60 min post treatment. Statistically significant (p-value ≤ 0.05) values compared for each time point to 0 min are indicated with an asterisk (*).

We carried out multivariate statistical analysis of the metabolomics datasets from untreated (0 min) *versus* CTCB405-treated (60 min) samples using MetaboAnalyst 5.0 to identify key differences. A partial least squares – discriminate analysis (PLS-DA, **Supplementary Figure 5A**) and variable importance in projection (VIP, **Supplementary Figure 5B**) plot identified the top 50 metabolites contributing to the effect of CTCB405 treatment on the cell lines. Interestingly, a significant decrease in the level of O-acetylcarnitine and increase in the amount of L-carnitine were observed (**Supplementary Figure 5B**; **Figure 3**). The trend was most notable for Lister 427 cells where O-acetylcarnitine dropped drastically within 5 min after addition of the inhibitor and then remained stable up to 60 min. This reduction in O-acetylcarnitine was mirrored by an increase of L-carnitine (**Figure 3C**). In EATRO 1125 cells, the decrease of O-acetylcarnitine occurred more gradually, while only a small transient increase in the L-carnitine levels was observed after addition of CTCB405 (**Figure 3D**). In contrast with *T. b. brucei*, relatively higher amounts of both O-acetylcarnitine and carnitine were found in *T. congolense* cells with no obvious change in their levels over time (**Figures 3A, B**).

Other major changes in the metabolome concerned glycerophospholipids, the major structural lipids in eukaryotic membranes, including phosphatidylcholine, phosphatidylethanolamine, and phosphatidylserine, as well as sphingomyelin; however, there was no consistent pattern of increase or decrease upon treatment (**Supplementary Figure 5B**).

## Discussion

**Figure 4:**
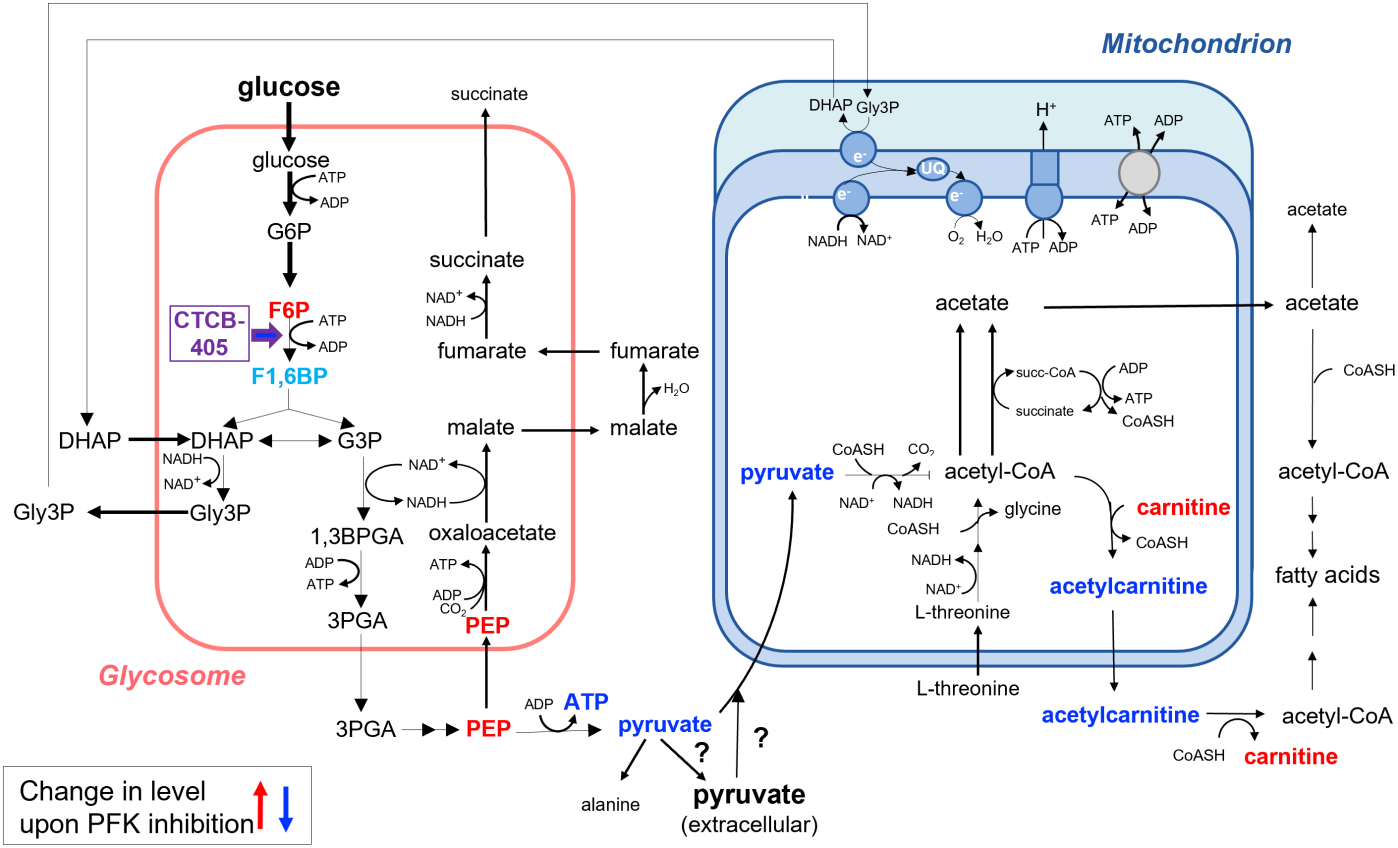
Summary of the changes in the metabolome of BSF *Trypanosoma brucei* upon inhibition of PFK as measured in total cell extracts. Addition of the PFK inhibitor CTCB405 led to accumulation of its upstream glycolytic intermediate F6P. Moreover, a significant increase in the level of L-carnitine and decrease of that of O-acetylcarnitine were observed, suggesting that acetyl-CoA can exit the mitochondrion by an acylcarnitine translocase system in addition to the pathway demonstrated previously involving the conversion of acetyl-CoA to acetate in the mitochondrial matrix, followed by exit of acetate through a (still hypothetical) shuttle system and the activity of a cytosolic AMP-forming acetyl-CoA synthetase (Mazet et al., 2013). Results presented in this paper also suggest that the decrease of ATP production by reducing the glycolytic flux may be compensated – in part and transiently – by an increased ATP formation in the mitochondrion as a result of an enhanced flux from pyruvate or threonine through the cyclic activity of acetate:succinate CoA-transferase and succinyl-CoA synthetase to acetate. Metabolites with measured increased cellular levels are highlighted in red, those with decreased levels in dark blue. F1,6BP is indicated in light blue. This metabolite was not detected in the dataset (**Supplementary Table S1**), but its level was likely decreased upon PFK inhibition, with additional metabolic consequences as discussed in the text. For further details about the metabolic network involved, see the text and the legend of **Supplementary Figure 1**.

This study showed that addition of sublethal concentrations of the trypanosomatid PFK specific inhibitor CTCB405 causes multiple effects in the metabolome of the bloodstream-forms of these parasites, already within the first hour after its addition. As expected, the glycolytic intermediate immediately upstream of the enzyme, F6P, accumulates and ATP levels decrease (**Figure 4**). However, we noticed that a rapid initial decrease of the ATP level is followed by a slight recovery of the level, that then remains relatively constant, most notable in the *T. b. brucei* Lister 427 cells (**Figure 1**). This behavior is reminiscent of observations in our previous research with lethal concentrations of different PFK inhibitors of the CTCB family, where the ATP level consistently dropped initially very quickly but then transiently increased significantly before gradually decreasing again resulting in parasite death (McNae et al., 2021). We hypothesize that the temporary increase in total cellular ATP results from an increased mitochondrial ATP production. The decreased ATP/ADP ratio in the cytosol due to inhibition of the glycolytic flux may be partially compensated by increased synthesis of ATP in the mitochondrion. An enhanced flux through the ATP-producing ASCT/SCS cycle may be enabled by an increased formation of acetyl-CoA, either derived from pyruvate when still being produced in sufficient amounts by glycolysis and/or from pyruvate or threonine taken up from the medium. However, the capacity of the mitochondrial ATP-producing system is not sufficient to fully compensate for the major drop in the amount of ATP formed by glycolysis when PFK is inhibited (**Figure 4**). Previously, it has been demonstrated that 30-50% inhibition of the glycolytic flux is sufficient to stop growth of BSF *T. brucei* (Haanstra et al., 2011). This explains why high CTCB405 concentrations only cause a temporary ATP increase (McNae et al., 2021), while at lower concentrations a partial compensation may be sustained for longer periods during which the carbon sources remain available, as observed in this work.

Surprisingly, the total ATP levels per cell detected for the two *T. b brucei* strains − the monomorphic Lister 427 and pleiomorphic EATRO 1125 − differed significantly, whereas very little variation was found for the four biological replicates of each strain (**Figure 1**). The cause remains to be determined. It may reflect different ATP/ADP ratios in cells of the two strains, maybe linked to slightly different metabolic rates. In this respect, it may be relevant that the growth rates of the strains also differed, although cultured in the same medium. Different cell size and/or different sizes of ATP-containing cell compartments may also contribute. Differences between the two strains were further detected in the three times higher dose of CTCB405 required for the EATRO 1125 cells than the Lister 427 trypanosomes to achieve a similar reduction in the population’s cell motility and the overall quantitatively less prominent changes in the EATRO 1125 metabolome upon treatment with these doses of the PFK inhibitor.

The increased L-carnitine and decreased O-acetylcarnitine levels following CTCB405 addition was another interesting observation. It strongly suggests that the mitochondrial membrane also contains a classical acylcarnitine translocase system by which acetyl-CoA can exit (**Figure 4**) in addition to the acetate shuttle proposed by Mazet et al. (2013). The CTCB405 induced changes in L-carnitine and O-acetylcarnitine levels supports the hypothesis of increased mitochondrial ATP production: the necessity for an increased flux through the ASCT/SCS cycle causes a shift of the intramitochondrial acetyl-CoA away from acetylcarnitine production, for exit *via* the translocase, in favor of the formation of acetyl-phosphate as reaction intermediate for ATP production by substrate-level phosphorylation.

The changes observed in the levels of different phospholipids upon treatment of BSF trypanosomes with CTCB405 are multiple and complex, and they differ between the three cell lines. A direct effect of the compound on lipid metabolism is very unlikely. BSF trypanosomes acquire their glycerophospholipids and fatty acids by two different routes: (i) an ATP-dependent uptake of LDL by receptor-dependent endocytosis and serum proteins such as albumin to which they are bound by fluid-phase endocytosis (Coppens et al., 1987; and reviewed by Van Hellemond and Tielens, 2006 and Smith and Bütikofer, 2010), and (ii) *de novo* synthesis from acetyl-CoA and glycerol 3-phosphate (Gilbert et al., 1983; Paul et al., 2001; Van Hellemond and Tielens, 2006; Smith and Bütikofer, 2010; Mazet et al., 2013). However, the relative contribution of both routes is still unknown and may vary dependent on external conditions. Inhibition of glycolysis will inhibit both processes by the decreased ATP, glycerol 3-phosphate, and acetyl-CoA production, but also other ATP-dependent processes required for cell growth such as protein expression. The measured levels of different lipids may thus have increased or decreased dependent on the relative extent by which lipid acquisition and cell growth were each affected by the CTCB405 induced drop in ATP supply. Alternatively or additionally, the partial decrease of ATP level may have affected the cell integrity; minor morphological changes were observed, as described above, during the incubation period at the inhibitor concentrations used. Such loss of integrity may have caused release of some lipids from membranes or lipid droplets which, during metabolite extraction, will have been retrieved.

Changes in levels of many other metabolites were observed after CTCB405 addition. Clear examples are certain fatty acids (**Supplementary Table 1** and **Figure S5B**). However, in none of the cases was a consistent pattern observed, and no explanation related to altered glucose catabolism could be found. We did not see any clear changes at early time points that might suggest direct off-target effects of CTCB405. We hypothesize that most of the changes are secondary due to the rapid drop in cellular ATP, since many cellular processes depend on ATP, either as a substrate or for regulation by kinases. Therefore, these changes have not been analysed further.

While CTCB405 caused several striking differences in the metabolome of BSF *T. brucei*, those in *T. congolense* were less prominent. This is attributed to the divergent metabolism between the two species (Steketee et al., 2021). The lower amounts of G6P, F6P and pyruvate in *T. congolense* compared to *T. brucei* may reflect differences in fluxes through the carbon metabolic network and/or different kinetic properties of enzymes involved. Additionally, due to the higher activity of the succinate shunt, converting PEP to succinate, less PEP becomes available for conversion into pyruvate in BSF *T. congolense*, while pyruvate is also not a metabolic end-product for these cells, but further metabolized within the mitochondrion at a much higher rate than in BSF *T. brucei*. Nonetheless, treatment with the PFK inhibitor caused accumulation of F6P and affected the motility and growth rate of the cells. This is in line with the previously reported essentiality of glucose catabolism by *T. congolense*, although glycolytic flux appears to occur at a considerably lower rate than in *T. brucei* (Steketee et al., 2021). A noteworthy difference between the two *Trypanosoma* species is that, at the CTCB405 concentrations used, no changes in L-carnitine and O-acetylcarnitine levels were observed in *T. congolense*. It is possible that the endogenous high activity of the mitochondrial acetate producing pathway under normal growth conditions allows for more ATP to be generated than required for the organelle’s maintenance, thus contributing to the cytosolic ATP pool, without a need to redistribute acetyl-CoA between efflux and flux through the ASCT/SCS cycle to enable it.

Interestingly, treatment of *T. brucei* Lister 427 with 0.25 μM CTCB405 and *T. brucei* EATRO 1125 and *T. congolense* IL3000 with 0.75 μM resulted in very similar reductions in parasite motility, while the effect on ATP levels was very different. Possible explanations could be that the trypanosomal flagellum has a separate pool of adenine nucleotides, with the availability of ATP to the flagellar beating machinery differing between the species. Alternatively, perhaps *T. congolense* prioritises maintaining a certain ATP level at the expense of other processes, in which case the effect on motility would be secondary and not a direct consequence of lack of ATP.

## Data availability statement

The original contributions presented in the study are included in the article/**Supplementary Material**. Further inquiries can be directed to the corresponding author.

## Author contributions

Conceptualization of the research: MW and PM. Experimental work: ZN, TM and KB. Data analysis: all authors. Supervision: AS and MW. Writing original draft: ZN, TM and PM. All authors contributed to the article and approved the submitted version.
