# Supplementary material for: Metabolic insights into phosphofructokinase inhibition in bloodstream-form trypanosomes
Source: Front Cell Infect Microbiol. 2023 Feb 14;13:1129791. doi: 10.3389/fcimb.2023.1129791 (PMC9971811; doi:10.3389/fcimb.2023.1129791)
Supplement: Supplementary file 1 [file DataSheet_1.docx]

**Supplementary Material**

1. **Materials and methods**
   1. **Trypanosome culturing**

BSF *Trypanosoma brucei* was grown in HMI-9 medium (Life Technologies) with 45 mM NaHCO_3_ and 256 μM β-mercaptoethanol, adjusted at pH 7.5 and filter sterilized, and supplemented with 10% (v/v) heat-inactivated fetal bovine serum (Thermo Fisher Scientific) and 1% (v/v) penicillin/streptomycin (10,000 U/ml) (Thermo Fisher Scientific). In addition, antibiotics required for maintenance of transgenic parasite cell lines were added: for *T. brucei* Lister 427 cells 2.5 μg/ml G-418 (Sigma-Aldrich) and for *T. brucei* EATRO 1125 2.5 μg/ml G-418, 5 μg/ml hygromycin (Calbiochem) and 0.1 μg/ml puromycin (Sigma-Aldrich). Cells were grown at 37 °C under water-saturated air with 5% CO_2_. Trypanosomes were counted using a hemocytometer, passaged to a density every two days, and maintained below a density of 1 x 10^6^ cells/ml.

BSF *T. congolense* was cultured in TcBSF3 medium, based on MEM medium (Sigma-Aldrich) with additions as described by Coustou et al. (2010), with a pH of 7.3. To 75% (v/v) filter-sterilized basal TcBSF3 medium were added 20% (v/v) goat serum (Sigma-Aldrich), 5% (v/v) tissue-grade sterile filtered water (Sigma-Aldrich), 0.0014% (v/v) β-mercaptoethanol, 200 mM glutamine and 1% (v/v) penicillin/streptomycin (10,000 U/ml) (Thermo Fisher Scientific). The *T. congolense* cells were grown without selectable marker, at 34 °C under water-saturated air with 5% CO_2_. Trypanosomes were counted using a hemocytometer every two days and the culture split to a density of 1 x 10^4^ cells/ml at each passage, and maintained below a density of 1 x 10^6^ cells/ml.

**1.2. Metabolomic profiling of trypanosomes**

**1.2.1. Metabolite extraction.** Metabolites were extracted from CTCB405-treated cultures of *Trypanosoma brucei brucei* strains Lister 427 and EATRO 1125 and *Trypanosoma congolense* strain IL3000, having equivalent densities of approximately 9 × 10^5^ cells/ml. To obtain 4 × 10^7^ cells per sample per time point, 45 ml of the culture was harvested by centrifugation at 1,250 ×g for 10 min. Supernatants were removed, retaining 5 μl of the spent media for metabolite extraction. The cell pellets were quenched in a 70% (v/v) ethanol/dry ice bath after a brief incubation on ice. Metabolites were extracted from the spent media and cell pellets by adding 200 μl of ice-cold chloroform/methanol/water (1/3/1 ratio) and mixing vigorously at 1,000 rpm for 1 h at 4 °C. The extraction mixtures were centrifuged at 13,000 ×g for 10 min, followed by collection of 180 μl of the supernatant into sterile 1.5 mm microcentrifuge tubes that were then stored at −80 °C until analysis.

**1.2.2. Data acquisition.** The untargeted metabolomics analysis was performed using liquid chromatography (LC) coupled to ion mobility (IM) quadrupole time of flight (qTOF) mass spectrometry (MS) as described previously (Pičmanová et al., 2022). The instrumentation consisted of an Agilent 1290 Infinity II series UHPLC system hyphenated with an Agilent 6560 IM-qTOF with a Dual Agilent Jet Stream Electron Ionization source. In brief, LC separation was performed on an InfinityLab Poroshell 120 HILIC-Z, 2.1 mm × 50 mm, 2.7 μm UHPLC column (Agilent Technologies 689775-924, Santa Clara, CA) coupled to an InfinityLab Poroshell 120 HILIC-Z, 3.0 mm × 2.7 μm UHPLC guard column (Agilent Technologies 823750-948). A 3.5 min gradient was run using two different solvent systems, one with low and the other with high pH. Data were acquired in the positive ionization mode using solvent A (10 mM ammonium formate in water with 0.1% formic acid, pH 3) and solvent B (10 mM ammonium formate in water/acetonitrile (1/9) with 0.1% formic acid, pH 3). Similarly, data were acquired in the negative ionization mode using solvent A (10 mM ammonium acetate in water, pH 9) and solvent B (10 mM ammonium acetate in water/acetonitrile (1/9), pH 9). The solvent gradient for positive and negative ionization modes consisted of 93% solvent B at the start of the run, which was reduced to 80% in 1.8 min, and further to 70% in 0.2 min, where it was maintained for 0.3 min. Subsequently, the column was returned to the initial condition of 93% solvent B at 2.35 min and maintained until 3.5 min. The column was kept at a constant temperature of 30 °C and a constant flow rate of 0.8 ml/min during the entire chromatographic separation. For analysis, 1 μl of sample was injected into the column. A pooled quality control sample was generated by combining equal volumes of each sample and injected five times at the beginning of the experiment to condition the column and after every five samples to monitor the instrument state over the course of data acquisition.

Data were acquired in the 50-1700 *m/z* range, with an MS acquisition rate of 0.8 scans/s for both ionization modes. The nebulizer pressure was set to 60 psi, gas temperature to 225 °C and drying gas (N_2_) flow rate to 13 l/min. Sheath gas was set to 340 °C with a flow rate of 12 l/min, and the instrument was operated at a capillary voltage of 3000 V, nozzle voltage of 200 V, fragmentor voltage of 395 V, and octupole voltage of 750 V. Instrument calibration and tuning was performed separately for each ionization polarity using the ESI-L low concentration tuning mix from Agilent Technologies. A reference mass solution consisting of 50 μM ammonium trifluoroacetate, 5 μM purine and 1.125 μM HP-0921 was injected continuously into each sample to recalibrate for accurate mass and drift time during data processing. The ES-TOF reference mass solution kit was purchased from Agilent Technologies (Santa Clara, CA).

**1.2.3. Data processing and statistical analysis.** The Agilent MassHunter 10.0 software suite was used for data acquisition and processing. Briefly, ion-mobility multiplexed data files and calibration files which were obtained from the MassHunter Data Acquisition 10.0 software were demultiplexed using the PNNL PreProcessor v2020.03.23. The default settings were applied to the data for demultiplexing, moving average smoothing, saturation repair, and spike removal. Data files were then recalibrated for accurate mass and drift time using the AgtTofReprocessUi and IM-MS Browser 10.0, respectively. Two reference masses, *m/z* 121.050873 and 922.009798 (positive ionization mode) and *m/z* 112.985587 and 1033.988109 (negative ionization mode) were used for recalibration.

Mass Profiler 10.0 was used to align and select molecular features with a retention time tolerance of ± 0.3 min, drift time tolerance of ± 1.5% and accurate mass tolerance of ± (5 ppm + 2 mDa). Additional peak deconvolution (May et al., 2020) was performed in the High Resolution Demultiplexer (HRdm) 1.0 beta v41 using the raw multiplexed data, the reconstructed demultiplexed data, and the Mass Profiler features list (.cef files). Molecular features were re-extracted from HRdm files using Mass Profiler 10.0 and annotated using accurate mass and CCS values with the McLean CCS Compendium PCDL (version 20191101; Pincache et al., 2018). A 10 ppm window was applied for *m/z* matches, a CCS value tolerance of ± 1% was applied, and the positive ion species (M+H)^+^, (M+Na)^+^ and the negative ion species (M-H)^−^ were searched. Annotated metabolites are therefore class II identifications, or class III for lipid species, as per the metabolite standards initiative (Sumner et al., 2007).

Multivariate statistical analysis was performed using the MetaboAnalyst 5.0 web-based platform (Chong and Xia, 2020). The data were log-transformed and pareto-scaled. Annotated molecular features were used to generate PLS-DA and VIP plots. Peak intensities were analyzed using MS-Dial (version 4.90) and used to generate the bar plots presented here.

1. **Supplementary figures**

**2.1. Glucose metabolism of bloodstream-form *Trypanosoma brucei***

**Figure S1. Current knowledge about aerobic glucose metabolism in bloodstream-form *T. brucei.*** The first seven enzymes of the glycolytic pathway are sequestered in peroxisome-related organelles called glycosomes. Glucose, taken up by the trypanosome from the blood, enters the glycosomes and is stepwise converted into 3-phosphoglycerate (3PGA) that exits the organelles. The last three steps, responsible for the conversion of 3PGA to pyruvate, occur in the cytosol. Inside glycosomes, the use of ATP by hexokinase and PFK and its formation by phosphoglycerate kinase are balanced. Net ATP production by pyruvate kinase occurs in the cytosol. NADH formed inside the glycosomes by glyceraldehyde-3-phosphate dehydrogenase is re-oxidized by a glycosomal NADH-dependent glycerol-3-phosphate dehydrogenase, an electron shuttle involving glycerol 3-phosphate (Gly3P) and dihydroxyacetone phosphate (DHAP) and a mitochondrial glycerol-3-phosphate oxidase system, comprising an FAD-dependent glycerol-3-phosphate dehydrogenase, ubiquinone and an alternative oxidase; this process is not coupled to mitochondrial ATP synthesis. Most pyruvate is excreted. Minor excreted end products from glucose metabolism can be succinate, alanine, and acetate. Part of the pyruvate may enter the mitochondrion where it is oxidized and decarboxylated to acetyl-CoA. The acetyl-CoA is converted into acetate, either by the combined activity of acetate:succinate CoA-transferase and succinyl-CoA synthetase A (ASCT/SCS cycle) with concomitant ATP formation or by an acetyl-CoA thioesterase. The names of the end products are boxed and given in white font against a red background. The thickness of the arrows representing reactions and the size of the boxes reflect the respective importance of the fluxes. Threonine, also taken up by the trypanosomes, may contribute to the acetate production; the path of this threonine metabolism is depicted in green. The light blue colored area of the mitochondrion represents the intermembrane space between the mitochondrial outer membrane (not drawn) and the inner membrane containing the respiratory complexes, the F_o_F_1_-ATP synthase and the ADP/ATP exchange transporter. For further details, see the main text and references cited therein. Enzymes are: 1, hexokinase; 2, glucose-6-phosphate isomerase; 3, phosphofructokinase; 4, aldolase; 5, triose-phosphate isomerase; 6, glyceraldehyde-3-phosphate dehydrogenase; 7, phosphoglycerate kinase; 8, phosphoglycerate mutase; 9, enolase; 10, pyruvate kinase; 11, NADH-dependent glycerol-3-phosphate dehydrogenase; 12, phosphoenolpyruvate carboxykinase; 13, malate dehydrogenase; 14, fumarase; 15, fumarate reductase; 16, alanine aminotransferase; 17, pyruvate dehydrogenase complex; 18, acetyl-CoA thioesterase; 19, acetate:succinate CoA-transferase; 20, succinyl-CoA synthetase; 21, L-threonine dehydrogenase; 22, 2-amino-3-ketobutyrate CoA-transferase; 23, AMP-forming acetyl-CoA synthetase; 24, NADH dehydrogenase; 25, FAD-dependent glycerol-3-phosphate dehydrogenase; 26, alternative oxidase; 27, F_o_F_1_-ATP synthase; 28, ADP/ATP carrier.

**2.2. Trypanosome motility assays**

**
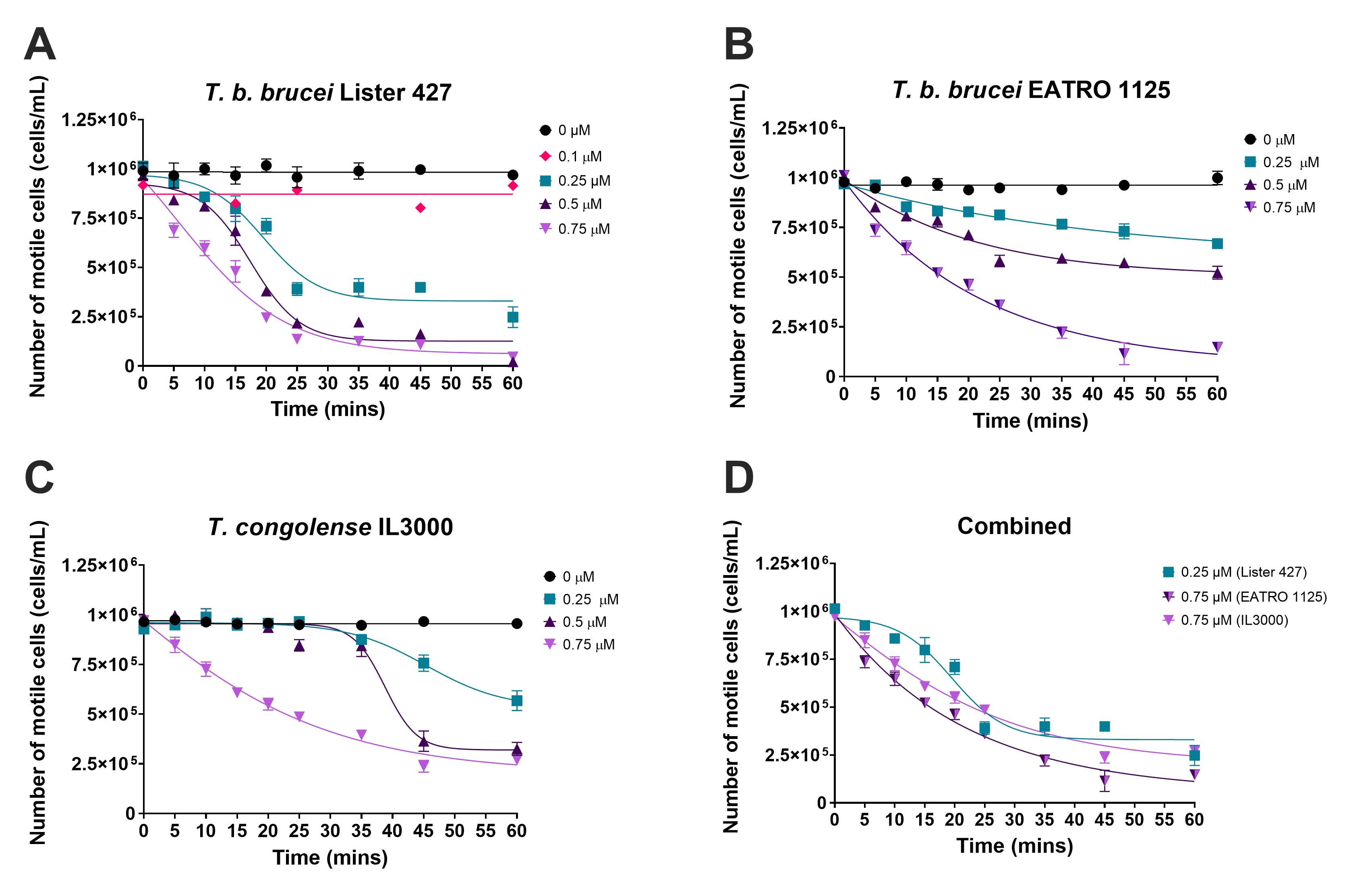
**

**Figure S2.** CTCB405 reduces cell motility in a concentration dependent manner in (**A**) *T. b. brucei* Lister 427, (**B**) *T. b. brucei* EATRO 1125 and (**C**) *T. congolense* IL3000. Cells were treated with 0, 0.1, 0.25, 0.5 and 0.75 μM CTCB405, and changes in motility determined as described in the main text of the paper, with the experiment done with two technical replicates for each strain. (**D**) Overlay of results from panels A, B and C.

**2.3. Conservation of the CTB405 binding pocket in *Trypanosoma* PFK**


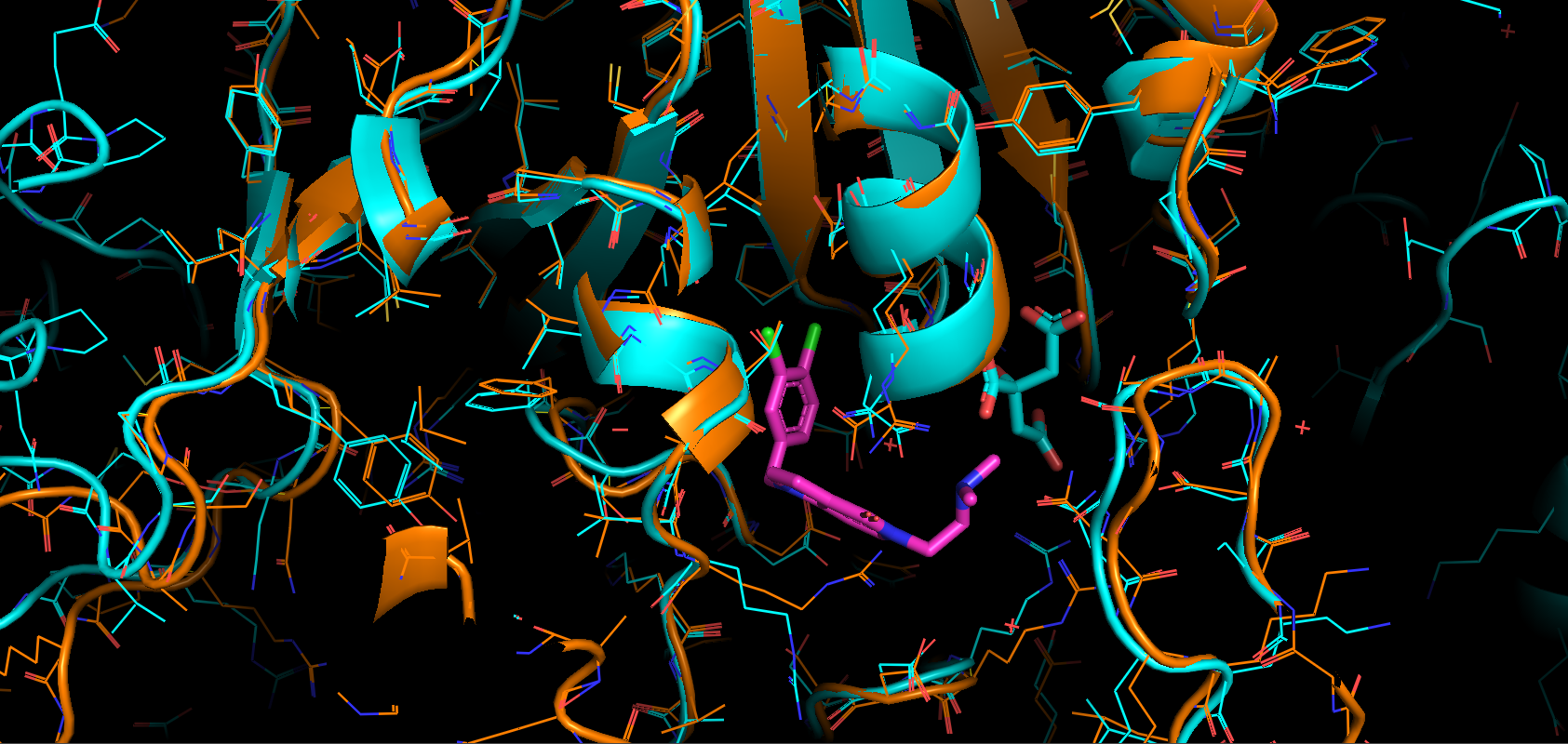


**Figure S3. The CTCB405 binding pocket of *T. brucei* PFK is fully conserved in *T. congolense* PFK.** A model structure of *T. congolense* PFK (orange) generated by AlphaFold (Jumper et al., 2021) fits the *T. brucei* PFK crystal structure (cyan) complexed with CTCB405 (pink) with an RMS value of 0.56 Å. All residues in the inhibitor-binding pocket are conserved between the enzymes of the two species. The structure of *T. brucei* PFK:CTCB405 was determined by McNae et al. (2021) and is available in the Protein Database under accession 6QU4. The *T. congolense* PFK amino-acid sequence used was taken from NCBI, accession code CCD13494.1.

**2.4. CellTiter-Glo® 3D ATP standard curve**

**
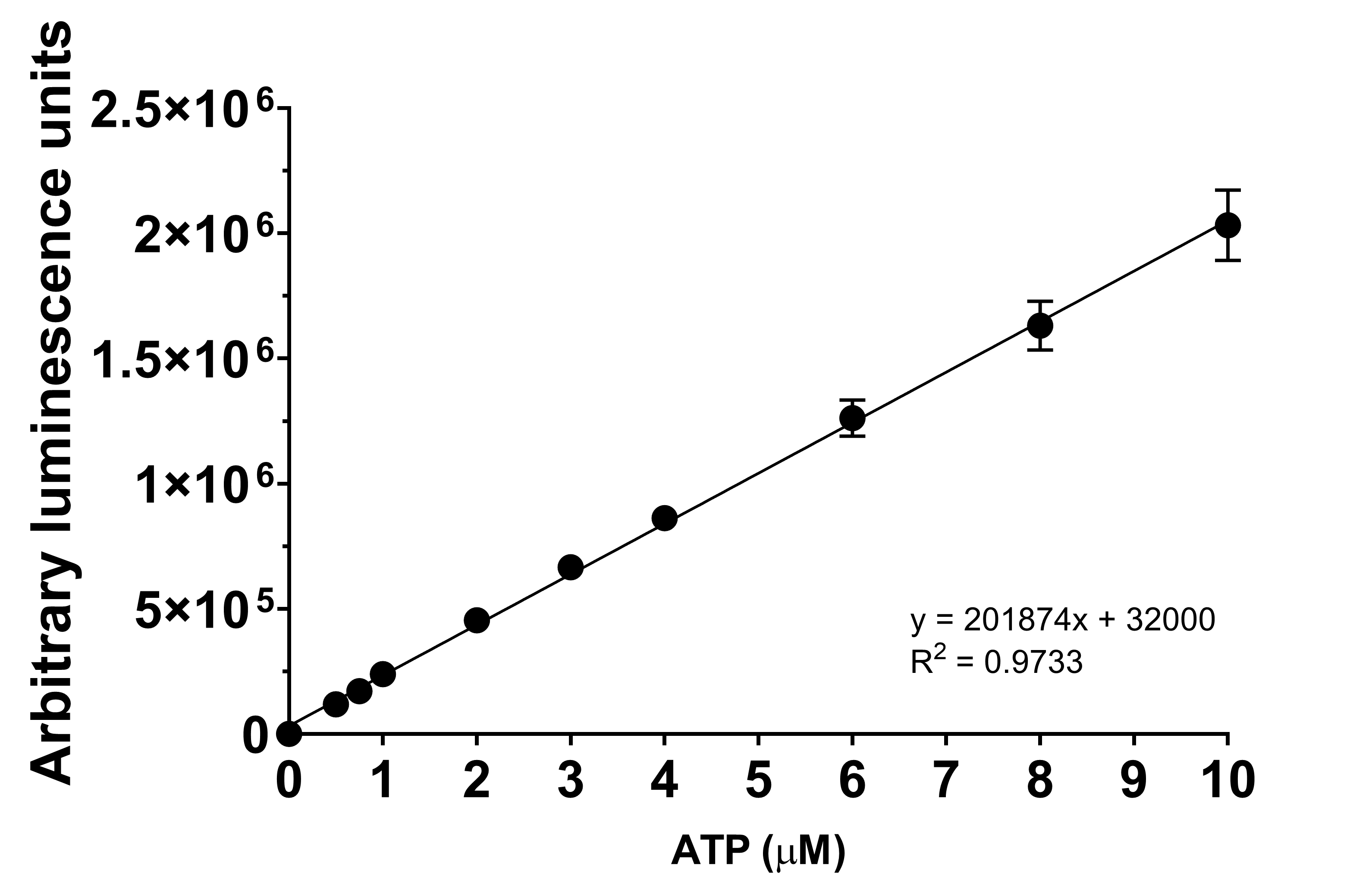
**

**Figure S4.** ATP standard curve for the CellTiter-Glo® 3D Assay, determined as described in the main text of the paper.

**2.5. Metabolome analysis of CTCB405 treated trypanosomes**


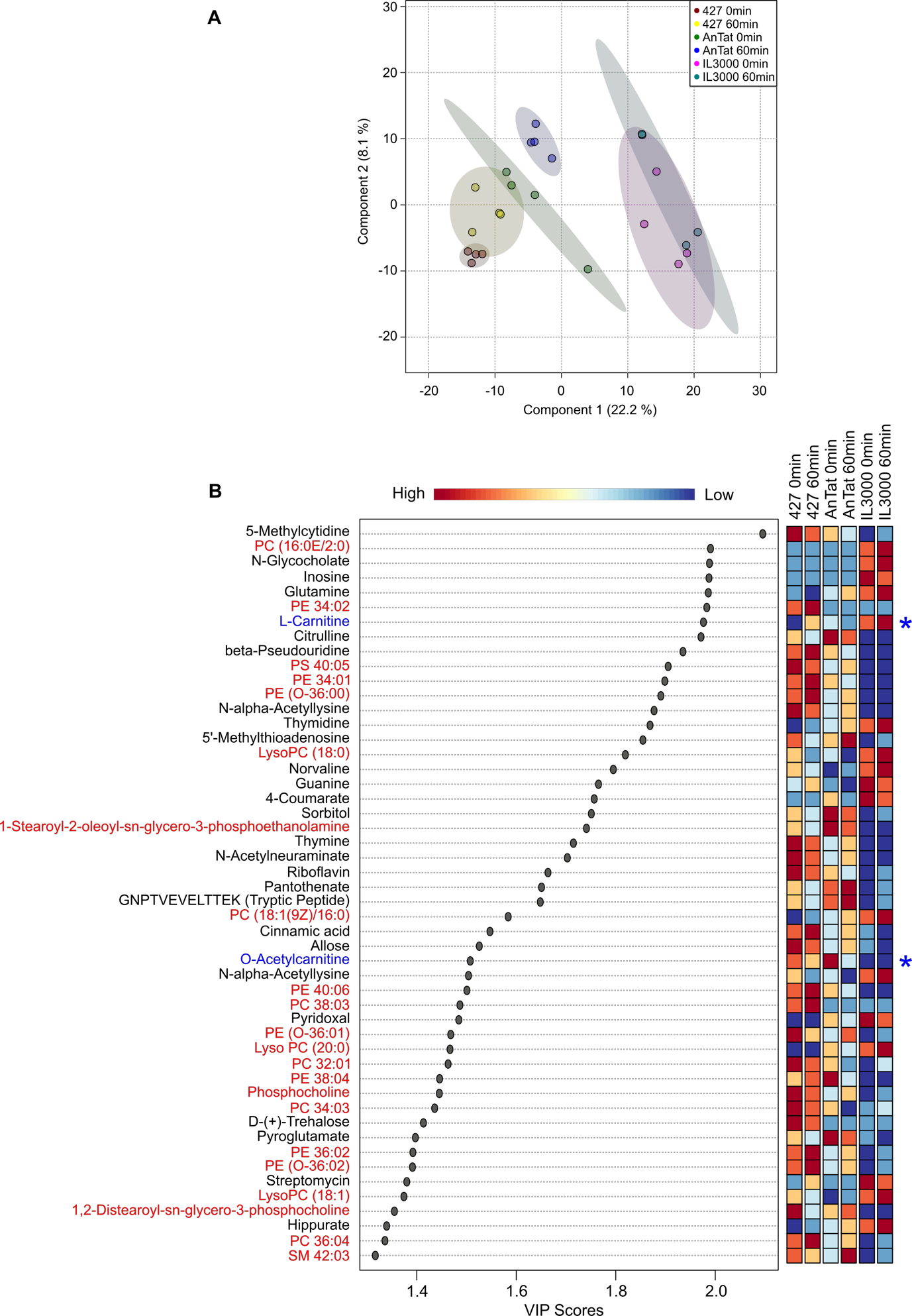


**Figure S5. Multivariate statistical analysis identifies metabolites contributing to the effect of CTCB405 treatment on BSF *T. b. brucei* Lister 427, *T. b. brucei* EATRO 1125 and *T. congolense* IL3000 cells.** (A) Partial least squares – discriminate analysis (PLS-DA) 2D score plot shows the separation of treated (t = 60 min) and untreated (t = 0 min) groups in all cell lines. Each colored dot corresponds to an individual sample and the shaded zone shows the 95% confidence interval for n = 4. (B) Variable importance in projection (VIP) plot shows the top 50 metabolites contributing to the separation of groups seen in PLS-DA plot. The heatmap shows the average of four replicates. L-carnitine and O-acetylcarnitine identified through this analysis are highlighted in blue font and are also indicated by asterisks. The glycerophospholipids, phosphatidylcholine (PC), phosphatidylethanolamine (PE), phosphatidylserine (PS), and sphingomyelin (SM) are highlighted in red font.

**3. Supplementary Tables**

Supplementary tables S1 and S2 are provided as separate files: Table S1.xlsx and Table S2.xlsx.

**Table S1: Raw output file obtained from the untargeted metabolomics analysis containing the annotated metabolites and their peak intensities in each sample.** The ion species identified, accurate mass and CCS value in the database are also provided. Also included are the average and standard deviation (SD) of the retention time (RT), drift time (DT), collision cross section (CCS) and accurate mass (m/z) obtained for the samples. This dataset was used to generate Figure S5.

**Table S2: Peak intensities of targeted metabolites investigated using MS-Dial (version 4.90). These data correspond to Figures 2 and 3.** Both accurate mass (m/z) and drift tube ion mobility parameters: mobility and collision cross section (CCS) were used to extract the peak height of these metabolites. Statistical significance calculations are also included.

**4. References**

Coustou, V., Guegan, F., Plazolles, N., Baltz, T. (2010) [Complete in vitro life cycle of Trypanosoma congolense: development of genetic tools.](https://pubmed.ncbi.nlm.nih.gov/20209144/) *PLoS Negl. Trop. Dis.* 4(3):e618. doi: 10.1371/journal.pntd.0000618

Jumper, J., Evans, R., Pritzel, A., Green, T., Figurnov, M., Ronneberger, O. et al. (2021) [Highly accurate protein structure prediction with AlphaFold.](https://pubmed.ncbi.nlm.nih.gov/34265844/) *Nature.* 596(7873), 583-589. doi: 10.1038/s41586-021-03819-2

May, J.C., Knochenmuss, R., Fjeldsted, J.C., McLean, J.A. (2020) [Resolution of Isomeric Mixtures in Ion Mobility Using a Combined Demultiplexing and Peak Deconvolution Technique.](https://pubmed.ncbi.nlm.nih.gov/32628451/) *Anal. Chem.* 92(14), 9482-9492. doi: 10.1021/acs.analchem.9b05718

McNae, I.W., Kinkead, J., Malik, D., Yen, L.H., Walker, M.K., Swain, C. et al. (2021) [Fast acting allosteric phosphofructokinase inhibitors block trypanosome glycolysis and cure acute African trypanosomiasis in mice.](https://pubmed.ncbi.nlm.nih.gov/33594070/) *Nat. Commun.* 12(1):1052. doi: 10.1038/s41467-021-21273-6

Pang, Z., Chong, J., Zhou, G., de Lima Morais, D.A., Chang, L., Barrette, M., et al. (2021) [MetaboAnalyst 5.0: narrowing the gap between raw spectra and functional insights.](https://pubmed.ncbi.nlm.nih.gov/34019663/) *Nucleic Acids Res.* 49(W1), W388-W396. doi: 10.1093/nar/gkab382

Picache, J.A., Rose, B.S., Balinski, A., Leaptrot, K.L., Sherrod, S.D., May, J.C., McLean, J.A. (2018) [Collision cross section compendium to annotate and predict multi-omic compound identities.](https://pubmed.ncbi.nlm.nih.gov/30774892/) *Chem. Sci.* 10(4), 983-993. doi: 10.1039/c8sc04396e

Pičmanová, M., Moses, T., Cortada-Garcia, J., Barrett, G., Florance, H., Pandor, S., Burgess, K. (2022) [Rapid HILIC-Z ion mobility mass spectrometry (RHIMMS) method for untargeted metabolomics of complex biological samples.](https://pubmed.ncbi.nlm.nih.gov/35229219/) *Metabolomics.* 18(3):16. doi: 10.1007/s11306-022-01871-1

Sumner, L.W., Amberg, A., Barrett, D., Beale, M.H., Beger, R., Daykin, C.A., et al. (2007) [Proposed minimum reporting standards for chemical analysis Chemical Analysis Working Group (CAWG) Metabolomics Standards Initiative (MSI).](https://pubmed.ncbi.nlm.nih.gov/24039616/) *Metabolomics.* 3(3), 211-221. doi: 10.1007/s11306-007-0082-2
